# Etiology and prognostic factors of sepsis among children and adolescents admitted to the intensive care unit

**DOI:** 10.5935/0103-507X.20150044

**Published:** 2015

**Authors:** Taís da Costa São Pedro, André Moreno Morcillo, Emílio Carlos Elias Baracat

**Affiliations:** 1 Department of Pediatrics, Faculdade de Ciências Médicas, Universidade Estadual de Campinas - Campinas (SP), Brazil.

**Keywords:** Sepsis/etiology, Prognosis, Child, Adolescent

## Abstract

**Objective:**

To determine the etiology and clinical disease progression variables of sepsis associated with the prognosis of patients admitted to a pediatric intensive care unit.

**Methods:**

Prospective and retrospective case series. Data were collected from the medical records of patients diagnosed with sepsis who were admitted to the pediatric intensive care unit of a general hospital from January 2011 to December 2013. Bacteria were identified in blood and fluid cultures. Age, sex, vaccination schedule, comorbidities, prior antibiotic use, clinical data on admission, and complications during disease progression were compared in the survival and death groups at a 5% significance level.

**Results:**

A total of 115 patients, with a mean age of 30.5 months, were included in the study. Bacterial etiology was identified in 40 patients. Altered peripheral perfusion on admission and diagnosis of severe sepsis were associated with complications. A greater number of complications occurred in the group of patients older than 36 months (p = 0.003; odds ratio = 4.94). The presence of complications during hospitalization was associated with death (odds ratio = 27.7). The main etiological agents were Gram-negative bacteria (15/40), *Staphylococcus aureus* (11/40) and *Neisseria meningitidis* (5/40).

**Conclusion:**

Gram-negative bacteria and *Staphylococcus aureus* predominated in the etiology of sepsis among children and adolescents admitted to an intensive care unit. The severity of sepsis and the presence of altered peripheral perfusion on admission were associated with complications. Moreover, the presence of complications was a factor associated with death.

## Abstract (pt)

**Objetivo:**

Determinar a etiologia e as variáveis clínicas e evolutivas da sepse associadas ao prognóstico nos pacientes internados em unidade de terapia intensiva pediátrica.

**Métodos:**

Série de casos prospectiva e retrospectiva. Coleta de dados nos prontuários de pacientes com diagnóstico de sepse internados na unidade de terapia intensiva pediátrica de hospital geral, de janeiro de 2011 a dezembro de 2013. Foram identificadas bactérias em culturas de sangue e líquidos biológicos. As variáveis idade, sexo, esquema vacinal, comorbidades, uso prévio de antibióticos, dados clínicos à admissão e complicações na evolução foram comparadas nos grupos sobrevida e óbito (nível de significância de 5%).

**Resultados:**

Foram incluídos115 pacientes, com média de idade de 30,5 meses. Etiologia bacteriana foi identificada em 40 pacientes. Perfusão periférica alterada à admissão e diagnóstico de sepse grave mostraram-se fatores associados às complicações. Houve maior número de complicações no grupo com idade maior de 36 meses (p = 0,003; *odds ratio* = 4,94). A presença de complicações durante a internação foi fator associado ao óbito (*odds ratio* = 27,7). As principais etiologias foram: bactérias Gram-negativas (15/40), *Staphylococcus aureus* (11/40) e *Neisseria meningitidis* (5/40).

**Conclusão:**

Bactérias *Gram*-negativas e *Staphylococcus aureus* predominaram na etiologia da sepse em crianças e adolescentes admitidos em terapia intensiva. A gravidade da sepse e a perfusão periférica alterada à admissão estiveram associadas às complicações. A presença de complicações foi fator associado ao óbito.

## INTRODUCTION

The progress in infectious disease control in recent decades, particularly in the prevention and adoption of new therapeutic measures, has had a significant impact on a relatively common clinical condition of sepsis in pediatrics. However, sepsis continues to be highly relevant in public health among both adults and children. The mortality rates among children affected by the most severe forms of the disease may reach values higher than 50% because of several factors, from poor vaccination coverage, difficulty of access to healthcare services and lack of intensive care hospital beds to the failure to adopt modern treatment protocols and new therapies. Even in developed countries, the mortality rates are not negligible (10 - 20%), and sepsis remains a leading cause of death among children.^(1-8)^

In intensive care units (ICU), the mortality rate from sepsis differs according to the clinical condition on admission and the development of septic shock and multiple organ dysfunction.^(9)^ Furthermore, the etiological agent of the disease and the presence of comorbidities affect the prognosis.^(10)^

Most children treated in emergency rooms and admitted to ICU with sepsis/septic shock show no bacterial isolation, which varies according to age, immune status and geographic location.^(11)^ The distribution of etiological agents changed considerably in the pediatric population during the post-vaccine era. A retrospective study conducted from 1998 to 2003 with children ages 2 months to 3 years in the United States showed, in the vaccinated cases, various etiological agents, including *Escherichia coli*, pneumococcal serotypes not present in the vaccine, *Staphylococcus aureus*, *Neisseria meningitidis, Salmonella* spp. and *Streptococcus pyogenes*.^(12)^

The ICU stay, with appropriate monitoring and treatment targeting the stabilization of clinical and laboratory parameters, is essential in the management of children with sepsis. The proper handling and initiation of vasoactive drugs in a timely manner combined with the monitoring and treatment of possible organ dysfunctions determine the disease progression and the presence or absence of complications and sequelae. In addition to treatment, other factors are associated with unfavorable progression of sepsis among intensive care patients, including the diagnosis time and host reaction responses to the infection.^(7,13)^

Epidemiological variables and clinical findings on admission also may be predictors of progression to multiple organ failure, complications or death. Identifying theses factors may contribute to improved and updated protocols for the diagnosis and treatment of sepsis. Furthermore, understanding the current etiological profile of sepsis, including the possible impact of vaccination programs, is essential to guide antibiotic therapy and reduce the risk of death.

The present study aimed to determine the etiology and disease progression variables of sepsis associated with its prognosis among patients admitted to a pediatric ICU.

## METHODS

This descriptive and observational cohort study with retrospective and prospective data was conducted in the pediatric ICU of *Hospital Municipal Dr. Mário Gatti* in the municipality of Campinas, São Paulo state (SP), Brazil. This general hospital provides care to patients referred from the basic healthcare network and provides urgent and emergency services to Campinas, with a reference population of 280,000, including 60,000 children younger than 15 years of age. No cancer or postoperative cardiac patients are admitted. The hospital has 10 pediatric ICU beds, averaging 265 admissions per year. The study was approved by the Research Ethics Committee of *Hospital Municipal Dr. Mário Gatti*. An informed consent form (Termo de Consentimento Livre e Esclarecido, TCLE) was signed by the children’s guardians in cases of prospective follow-up. Exemption from the TCLE requirement was requested in cases of retrospective follow-up. The project was submitted to and approved by the Brazil Platform (CAAE 31441614.1.0000.5482).

All children from birth to 15 years of age with a diagnosis of sepsis who were admitted to the pediatric ICU in 2011, 2012 (retrospective) and 2013 (prospective) were included in the study. Sepsis was defined as the documented or suspected infection associated with clinical and laboratory criteria indicative of the disease, according to the sepsis consensus definition established in Barcelona in 2005.^(1)^ Children with an underlying disease that could cause immunity changes (e.g., primary immunodeficiency and extreme prematurity) were excluded from the study.

The following variables were analyzed: sex (male/female), age (in months and by dividing the children into the age groups younger than 3 months, 3 - 12 months, 12 - 36 months and > 36 months for data analysis), clinical data on admission (systolic blood pressure, heart rate, and respiratory rate as normal, decreased or increased according to the values defined for age; altered capillary refill time if longer than 3 seconds; presence or absence of an altered level of consciousness), classification of disease severity on admission into sepsis or severe sepsis,^(1)^ the presence of a comorbidity, prior antibiotic use (yes/no), complete vaccination schedule (three doses of pneumococcal vaccine 10 and two doses of meningococcal vaccine C), presence or absence of complications during hospitalization, sequelae and death. Cultures were collected from blood and other body fluids. Data collection was conducted through consultation of medical records.

Comorbidity was defined as the existence of disease prior to admission to the pediatric ICU that was unrelated to sepsis (i.e., genetic, neurological, cardiac or pulmonary diseases). Complication was defined as any unexpected medical condition that would occur during the period of hospitalization in association with the disease that led to sepsis, the progression of sepsis itself or to procedures performed in the pediatric ICU.

The data were processed using the software Statistical Package for the Social Sciences (SPSS) 16.0 (SPSS Inc., Chicago, Illinois, USA) and Epi Info 6.04d (WHO, Geneva, Switzerland).

The Student’s *t*-test post-Blom transformation was used to compare the means of age regarding sex, sepsis severity, complications and death. The chi-square test or Fisher’s exact test were used to evaluate the association between the qualitative variables. The odds ratio (OR) and 95% confidence intervals (CI) of death for the variables sex, age, comorbidities, vaccination schedule, prior antibiotic use, blood pressure, capillary refill time, disease severity and complications and of complications for the variables age, blood pressure and capillary refill time were determined. A 5% significance level (α = 0.05) was adopted in all cases.

## RESULTS

Sepsis was diagnosed in 118 patients (14.9%), and 3 patients were excluded (1 with primary immunodeficiency and 2 with extreme prematurity). Of the 115 patients included in the study, 86 were allocated to the retrospective group and 29 to the prospective group. Most patients (77.4%) were referred from the basic healthcare network or were walk-ins, 13% were referred from the pediatric ward of *Hospital Municipal Dr. Mário Gatti*, and 9.6% were referred from urgent care centers. The general characteristics of patients admitted to the pediatric ICU in the study period are outlined in table 1. The age distribution in relation to sex, disease severity and the presence of complications is outlined in table 2.

**Table 1:** General characteristics of patients admitted to a pediatric intensive care unit

**Characteristic**	**Total patients (N = 790)**	**Patients with sepsis (N = 115)**
Mean age (months)	39.5 ± 53.7	30.5 ± 44.3
Age group (months)		
< 12	456 (57.8)	63 (54.8)
12 - 36	107 (13.5)	21 (18.3)
> 36	227 (28.7)	31 (26.9)
Sex		
Male	455 (57.6)	73 (63.5)
Female	355 (42.4)	42 (36.5)
Comorbidities		28 (24.3)
Genetic syndrome	-	2 (1.7)
Prematurity*	-	12 (10.4)
Neurological disease**	-	4 (3.5)
Pneumopathy***	-	9 (7.8)
Chronic renal failure	-	1 (0.9)
Without complications	-	83 (72.1)
Complications		32 (27.9)
Pleural effusion	-	9 (7.8)
MODS	-	5 (4.3)
Kidney failure	-	4 (3.5)
Abscess	-	4 (3.5)
Reversed CPA	-	3 (2.6)
Pneumothorax	-	2 (1.7)
Pancreatitis	-	1 (0.9)
Aseptic meningitis	-	1 (0.9)
DIC	-	1 (0.9)
Joint effusion	-	1 (0.9)
Heart failure	-	1 (0.9)
Death	38 (4.8)	15 (13.0)

MODS - multiple organ dysfunction syndrome; CPA - cardiopulmonary arrest; DIC - disseminated intravascular coagulation.

* longer than 28 weeks;

** epilepsy and cerebral palsy;

*** wheezy infant. Results are expressed as numbers (%).

**Table 2:** Age distribution (in months) of patients with sepsis admitted to a pediatric intensive care unit

	**Median**	**Interquartile range**	p value*
Sex			
Male	7.0	1.3 -28.8	0.225
Female	13.2	2.5 -68.4	
Disease severity			
Severe sepsis	10.4	2.3 -45.4	0.006
Sepsis	1.3	0.7- 9.0	
Complications			
Yes	25.1	7.2 -78.6	0.004
No	5.3	1.3 -26.2	

* probability according to the chi-square test.

Regarding the clinical variables on admission to the pediatric ICU, altered perfusion and diagnosis of severe sepsis were associated with complications (Table 3). A higher number of complications occurred in the group older than 36 months compared to the age groups 12 - 36 months, 3 - 12 months and younger than 3 months (p = 0.003) (Table 3).

**Table 3:** Comparison between the presence and absence of complications in patients with sepsis admitted to a pediatric intensive care unit

	**With complications**	**Without complications**	p value*	**OR**	**95%CI**
Age (months)					
< 3	6 (14.3)	36 (85.7)		1.00	
3 - 12	6 (28.6)	15 (71.4)	0.191	2.40	0.56 - 10.35
12 - 36	6 (28.6)	15 (71.4)	0.054	4.80	0.89 - 28.56
> 36	14 (45.2)	17 (54.8)	0.003	4.94	1.44 - 17.69
Blood pressure					
Decreased	4 (33.3)	8 (66.7)	0.732	1.37	0.38 - 4.99
Normal/Increased	24 (26.7)	66 (73.3)			
Capillary refill					
Altered	27 (36.0)	48 (64.0)	0.007	3.94	1.27 - 13.00
Normal	5 (12.5)	35 (87.5)			
Disease severity					
Severe sepsis	32 (31.1)	71 (68.9)	0.019	-	-
Sepsis	0 (0.0)	12 (100.0)			

OR - odds ratio; 95%CI - 95% confidence interval;

* probability according to the chi-square test or Fisher's exact test. Results are expressed as numbers (%).

The mortality rate was 13% (15/115) and was highest in the age group from 12 to 36 months (23.8%). No significant differences were found for sex, age, presence of comorbidities, vaccination schedule and prior antibiotic use when comparing survival and death among the groups. Among the clinical variables, the presence of complications during hospitalization was associated with death (OR = 27.7; p < 0.001) (Table 4).

**Table 4:** Comparison between death and survival in patients with sepsis admitted to a pediatric intensive care unit

	**Death**	**Survival**	p value*	**OR**	**95%CI**
Sex					
Male	7 (9.6)	66 (90.4)	0.147	0.45	0.13 - 1.52
Female	8 (19)	34 (81)			
Age (months)					
< 3	3 (7.1)	39 (92.9)		1.00	
3 - 12	3 (14.3)	18 (85.7)	0.391	2.17	0.31 - 15.38
12 - 36	5 (23.8)	16 (76.2)	0.104	4.06	0.72 - 24.97
> 36	4 (12.9)	27 (87.1)	0.448	1.93	0.33 - 12.2
Comorbidities					
Yes	6 (21.4)	22 (78.6)	0.192	2.36	0.66 - 8.36
No	9 (10.3)	78 (89.7)			
Vaccination schedule					
Incomplete	9 (4.3)	75 (89.3)	0.227	0.50	0.14 - 1.78
Complete	6 (15.2)	25 (80.6)			
Prior antibiotic use					
Yes	1 (4.3)	22 (95.7)	0.297	0.25	0.01 - 2.03
No	14 (15.2)	78 (84.8)			
Blood pressure					
Decreased	2 (16.7)	10 (83.3)	0.630	1.6	0.31 - 8.37
Normal/Increased	10 (11.1)	80 (88.9)			
Capillary refill					
Altered	13 (17.3)	62 (82.7)	0.061	3.98	0.79 - 27.11
Normal	2 (5.0)	38 (95.0)			
Disease severity					
Severe sepsis	15 (14.6)	88 (85.4)	0.360	-	-
Sepsis	0 (0.0)	12 (100.0)			
Complications					
Yes	13 (40.6)	19 (59.4)	< 0.001	27.71	5.23 - 195.3
No	2 (2.4)	81 (97.6)			

OR - odds ratio; 95%CI - 95% confidence interval;

* probability according to the chi-square test or Fisher's exact. Results are expressed as numbers (%).

Among the 115 patients, 40 cultures were positive (34.8%). Most positive tests occurred in blood cultures, followed by urine cultures (Table 5). The most common community-acquired infectious agents were *S. aureus* (11/40), *Klebsiella pneumoniae *(7/40), *N. meningitidis* (5/40), *Pseudomonas aeruginosa* (4/40) and *E. coli* (4/40). The other agents found included *Streptococcus pneumoniae *(2), *S. pyogenes *(1), *Serratia marcescens *(1), *Enterococcus faecalis* (1), *Staphylococcus haemolyticus* (1) and *Enterobacter aerogenes* (2); the last two were detected in patients from the ward.

**Table 5:** Bacterial agents isolated in positive cultures from patients with sepsis admitted to a pediatric intensive care unit

**Positive cultures**	**Blood culture**	**Urine culture**	**Cerebrospinal fluid**	**Pleural fluid**	**Abscess**
*Staphylococcus aureus*	11	-	-	1	-
*Streptococcus pneumoniae*	-	-	-	1	1
*Klebsiella pneumoniae*	5	3	1	-	-
*Escherichia coli*	-	4	-	-	1
*Pseudomonas aeruginosa*	3	2	-	-	-
*Streptococcus pyogenes*	1	-	-	-	1
*Neisseria meningitidis*	4	-	3	-	-
*Serratia marcescens*	1	-	1	-	-
*Escherichia coli ESBL*	-	2	-	-	-
*Enterococcus faecalis*	-	1	-	-	-
*Staphylococcus haemolyticus*	1	-	-	-	-
*Enterobacter aerogenes*	2	1	-	-	-
Total	28	13	5	2	3

## DISCUSSION

The sample analyzed included 63.5% male patients, a number quite similar to the 2011 study^(7)^ and similar to other previous studies.^(14-18)^ The age analysis of this study’s patients showed they were mostly younger than 12 months (54.8%) and younger than 36 months in 73% of cases, which is similar to most studies.^(15-17,19)^

The presence of comorbidities among the study’s children was small (24.3%) compared to the literature, which reports percentages ranging from 40 to 91%.^(7,11,14-17,19)^ This finding reflects the characteristics of the setting in which the study was conducted, which was a pediatric ICU with a general profile of admissions for community-acquired clinical diseases.

The older mean age among patients with severe sepsis has not been reported in the literature. Possible variables, including time from disease onset to admission or the difficulty in recognizing the clinical course of sepsis in older children, who usually maintain a preserved neurological status longer, may have contributed to this finding.

The main factor associated with death (OR = 27.7; p < 0.001) in the present study was the presence of complications during hospitalization. This finding urges an early therapeutic approach in patients who show that type of progression during hospitalization. The complications reported in this study show the clinical severity of those cases wherein various organs and systems are affected by infection or the inflammatory/infectious process has progressed extensively at the primary site, with an insufficient or late defense response.

Altered perfusion and the diagnosis of severe sepsis on admission were factors associated with complications, and progression with complications was a predictor of death, suggesting that the establishment of an early and rapid treatment of sepsis in the emergency department is key. Patients with delayed diagnosis who are in the early stages of treatment are admitted to the pediatric ICU already with clinical signs of peripheral circulatory dysfunction (elevated capillary refill time) or impairment of two or more organs, which are characteristic of severe sepsis. This clinical profile progresses more frequently with complications and, consequently, death. This association was also reported by Shime et al., who observed increased mortality rates in patients with signs of septic shock on admission to the pediatric ICU.^(19)^

The present study identified factors associated with complications and death in pediatric patients with sepsis admitted to the pediatric ICU of a general hospital. Furthermore, the study sought to identify the etiological agents in blood cultures and other body fluids, which could substantiate or refute the current discussion on the empirical use protocols of antibiotics in the initial stages of treatment of sepsis in children.

Despite the low positivity of cultures (34.8% of cases), the most prevalent pathogen was *S. aureus*, confirming what recent studies indicate to be a changing trend in the etiological profile of sepsis in pediatrics since the introduction of the pneumococcal and meningococcal vaccines.^(20)^ Several studies conducted in different parts of the world also identified *S. aureus* as the main etiological agent in cultures from children admitted to a pediatric ICU with a diagnosis of sepsis,^(8,11,14-17)^ including the methicillin-resistant *S. aureus* (MRSA).^(19)^ That finding would have a clinical implication on the empirical antibiotic treatment of a patient with sepsis. The introduction of antistaphylococcal antibiotic therapy should be considered a priority in those situations, where the presence of *S. aureus* in the skin, soft tissues, bones, joints and pericardium is more common.

Considering community-acquired bacteria, four of the five cases of positive culture for *N. meningitidis* occurred in children older than 3 years. Despite their age, those patients had an incomplete vaccination schedule for meningococcal C and, therefore, increased susceptibility to meningococcal disease. The incidence of infections by *N. meningitidis* appears to show a downward trend in the studies, which could be a consequence of vaccination conducted in children from 3 months of age. A U.S. multicenter study conducted from 1995 to 2005 confirms this trend. In 1995, the rate of that pathogen among children with sepsis was 1.2%, followed by 0.7% in 2000 and 0.4% in 2005.^(17)^ Australia and New Zealand data from a multicenter prospective study analyzed at five-year intervals showed a significant decrease in meningococcal incidence in cases of sepsis and septic shock (from 13.8% in the period from 2002 to 2007 to 6.4% from 2008 to 2013).^(8)^

*S. pneumoniae*, one of the main etiological agents in children during the pre-vaccine era, was the sixth most prevalent etiological agent in this study (5% positive blood cultures). The prevalence observed is similar to the literature, which reports values from 2.1 to 11.8%.^(7,8,11,15,16,19)^

It is noteworthy that the bacteria identified in the cultures are uncommon etiological agents of community-acquired sepsis in pediatrics. *E. faecalis, S. haemolyticus *and* E. aerogenes *were identified in patients from the hospital general ward who had been hospitalized for at least 24 hours, which may suggest a potential hospital-acquired infection. *E. coli* with extended-spectrum beta-lactamase (ESBL) was isolated from the urine of patients with a vesicostomy, which increases the risk of acquiring resistant strains of the bacterium.

The mortality rate (13%) observed in this study was within the values found in the literature (8.8% to 56.1%) and was 23.8% in the age group from 12 to 36 months.^(7,14-17)^ A Brazilian study reviewing data from the Hospital Information System of the Unified Health System (Sistema de Informações Hospitalares do Sistema Único de Saúde, SIH-SUS) found a mortality rate from sepsis, in the period from 1992 to 2006, ranging from 19.7 to 20.5%, which was highest in the age group from 1 month to 1 year.^(18)^ A Japanese study observed an 18.9% overall mortality from sepsis, including 25% in adolescents, 26% in children and 14% in infants,^(19)^ in contrast to other studies that show a higher mortality rate among infants.^(16,17)^ A factor that may have contributed to that difference in age groups is that the vaccination schedule begins at different times in each country.

The main study limitations were the small number of cases compared to sepsis studies in adults and the inclusion of patients from a single healthcare provider, which has a well-defined demand that is restricted to healthcare services of the municipal healthcare network. The inclusion of other settings that would include tertiary referral hospitals and centers with regional coverage could bring additional information and identify other prognostic factors in sepsis among children. Furthermore, the low positivity and lack of standardization in culture collection time may have affected the results of etiological identification.

The study results indicate the need for a prospective study of patients admitted to the ICU of general hospitals, preferably a multicenter study that includes the timely collection of cultures in the first hour of sepsis treatment.

## CONCLUSION

In this study population, *S. aureus* and Gram-negative bacteria predominated as etiological agents in the group of patients diagnosed with sepsis who were admitted to an intensive care unit. The severity of sepsis and altered peripheral perfusion on admission were associated with complications during disease progression. Moreover, the presence of complications during hospitalization was a factor associated with death.
